# Reversible Information of Encrypted Image Based on Feature Difference Detection and Wavelet Transform

**DOI:** 10.1155/2021/5483001

**Published:** 2021-12-21

**Authors:** Yongsheng Ding, Yunbo Wei, Shuisheng Zhang, Shihang Yu

**Affiliations:** School of Science, Qiqihar University, Qiqihar, Heilongjiang 161006, China

## Abstract

Aiming at the shortcomings of the existing lossless digital watermarking algorithm based on frequency domain in reversibility and embedding capacity, this study proposes a lossless digital image watermarking algorithm based on fractional wavelet transform, which is used for large-capacity reversible information hiding of images. First, the image is transformed by LeGall5/3 fractional wavelet, and then, the watermark is embedded in the high-frequency subband by the histogram shift method. In order to obtain maximum embedding capacity and reduce image distortion, the methods of selecting embedding parameters and stopping parameters are proposed, respectively. At the same time, in order to prevent overflow and reduce additional information, a new method of generating position map is proposed. The experimental results show that Lena is the result of multilayer embedding based on the algorithm in this study. In order to better observe the distortion phenomenon and enlarge the image, the Lena test image is the watermark image obtained after two and three layers of embedding, and its embedding capacity can be 2.7 bpp. It is proved that wavelet transform is suitable for encrypted images to implement covert communication.

## 1. Introduction

Image reversible information hiding technology refers to the technology of embedding additional hidden information in digital images and recovering the original images 100% after information extraction. This technology can be used in medical, military, judicial, and other fields that need to recover the original images without loss, so as to realize copyright protection and integrity certification [1] of images. Reversible information hiding technology can be divided into three categories: (1) lossless image compression technology; (2) based on difference expansion technology; (3) translation technology based on histogram. At present, algorithms are mainly divided into spatial domain and frequency domain algorithms. Representative spatial domain algorithms, such as those based on difference extension and histogram translation, have larger embedding capacity, lower quality of hidden graphs, and lower operating efficiency, while the latter has the advantages of simple operation and preestimation of embedding capacity. With the increasing number of leaks, the protection of information security and personal privacy has aroused widespread concern. Information owners are unwilling to directly expose sensitive data including personal privacy to open channels or unreliable databases. Therefore, information owners will first encrypt the original information by using encryption technology and then send or transmit ciphertext information [2]. For a channel or database administrator, additional information such as tag information and authentication information need to be directly embedded into ciphertext information for management. For example, the hospital database administrator will directly embed patient information and date information into the encrypted medical image and then save the encrypted information into the database. Obviously, when needed, original image should be completely restored [3].

Digital image, which contains abundant user information, has become an indispensable means of interaction among countries and fields. At present, in the process of user information communication, images are mostly transmitted under unauthorized network environment, which brings huge hidden dangers to image information. Therefore, it is very important to keep the image information safe and confidential. In order to improve the security of image transmission, scholars put forward corresponding digital image encryption technologies, which are mainly divided into two categories: global encryption and selective encryption. Global encryption technology is to diffuse all pixels in the image [4]. Kavitha K.J. designed an image encryption algorithm combining self-coding, chaos, and hyperchaotic. By improving discrete Henon mapping parameters, the random sequence is generated by disturbing the regression coding and logistic chaotic map, which is fused with the sequence output by the improved 3D Lorenz reversible map, and the diffusion mechanism is constructed to complete the plaintext encryption [5]. Zhang designed the image encryption technology based on row and column transformation, formed the row and column transformation engine by iterative Logistic mapping, and built the corresponding encryption mechanism to complete the encryption of the input image. Experimental results show that the algorithm has ideal encryption speed and security [6]. Nasrallah N. Min Ming designed the image encryption technology based on the wavelet transmission and chaotic system. By iterating the chaotic system and outputting random sequence, the source point of wavelet transmission was determined. Rood operation was introduced to scramble plaintext, and the encryption model was built by relying on plaintext content and key [7]. Although the algorithm in literature has good performance, the information embedding side can only embed the information in the space reserved before encryption. Once the block information is leaked, the embedded information is easy to be extracted and replaced, which poses a great threat to the security of the embedded information and the carrier image. Considering embedding capacity and algorithm security, a reversible information hiding algorithm based on wavelet transform is proposed in this study. First, the plaintext image is divided into blocks, and then, two adjacent pixels are divided into nonoverlapping pixel groups. According to the transformation key, the corresponding pixel group is selected for integer transformation. The two adjacent pixels after transformation are constrained by integer transformation, so that the original pixel group can still be restored after any LSB is replaced. Therefore, the transformed pixel groups in the ciphertext image are found according to the transformation key, and the information hider uses the embedding key to generate a random sequence to determine the position of information embedding in each pixel group and replaces one of the LSB to complete the embedding of hidden information. Embedding information can be extracted correctly only when both transform key and embedding key are possessed. The original image can be restored only when both transformation key and encryption key are possessed.

## 2. Wavelet Transform and Information Hiding Technology in Plaintext Domain

An information hiding algorithm based on decimal transformation is proposed. The algorithm takes two adjacent pixels as a group and performs decimal transformation. A brief introduction to wavelet transform technology is provided. Wavelet transform can decompose the image in multiresolution, and each wavelet transform will get 4 frequency subgraphs, which are LL (low-frequency subgraph), HL (high -requency subgraph in vertical direction), LH (high-frequency subgraph in horizontal direction), and HH (high-frequency subgraph). Among them, LL subgraph concentrates most of the energy of the image and is the approximation subgraph of the original image. Higher wavelet classification can be carried out to obtain the four frequency subgraphs of the second level (LL2, HL2, LH2, and HH2), and the middle high-frequency subgraph represents the edge and texture information of the image. Finally, the original image can be restored by inverse transformation. Specific characteristics of decimal transformation and its corresponding information hiding algorithm are as follows [8].

### 2.1. Wavelet Transform

Let *x*_1_ and *x*_2_ be the two adjacent pixels of 8-bit gray image, respectively, and the corresponding decimal values after transformation *y*_1_ and *y*_2_ can be obtained by the following formula.

(1) $$\begin{array}{c} y_{1}=\left(n+1\right)x_{1}-nx_{2},\\ y_{2}=-nx_{1}+\left(n+1\right)x_{2},n=1,2,\ldots . \end{array}$$

Furthermore, the transformed pixels *y*_1_, *y*_2_ meet

(2) $$\begin{array}{c} \left(n+1\right)y_{1}+ny_{2}=0\mathrm{mod}\left(2n+1\right),\left(n+1\right)y_{2}+ny_{1}=0\mathrm{mod}\left(2n+1\right),n=1,2,\ldots , \end{array}$$

if *y*_1_, *y*_2_ meet

(3) $$\begin{array}{c} y_{1}>0\text{ and }y_{1}+2n<255,0<y_{2}<255. \end{array}$$

Then, *y*_1_ and *y*_2_ replace *x*_1_ and *x*_2_, where the pixels will not overflow due to transformation and information embedding, so they can be used for embedding information. Otherwise, the original pixel value is left unchanged, and its position is marked by a binary matrix R.

### 2.2. Information Embedding

Insert additional information *a* into *y*_1_. The embedded pixel is ${\tilde{y}}_{1}=y_{1}+a$, and *y*_2_ remains the same. When *a*=0, the embedded two-pixel values still satisfy formula (2). If *a* ∈ [1,2*n*] is embedded, it is no longer satisfied. Formula (4) is obtained by substituting ${\tilde{y}}_{1}$ and *y*_2_ into formula (2).

(4) $$\begin{array}{c} \left(n-1\right){\tilde{y}}_{1}+ny_{2}=\left(n+1\right)a\mathrm{mod}\left(2n+1\right). \end{array}$$

With regard to ∀*a* ∈ [0,2*n*], the formula (4) has a unique result *c*=(*n*+1)*a*mod(2*n*+1). As when *a*=1, *c*=(*n*+1). Therefore, according to *c* value, restore the corresponding *a*. When embedding information, first find the untransformed pixel group (*x*_1_, *x*_2_) and then calculating (*n*+1)*x*_1_+*nx*_2_=*c* − mod(2*n*+1), *c* − ∈[0,2*n*]. By adding a number *b*, *y*_1_=*x*_1_+*b* and *y*_2_=*x*_2_ satisfy formula (2), where $b=\left(2n+1-\tilde{a}\right)\mathrm{mod}2n+1,b\in \left[0,2n\right]$, and *b* is called auxiliary information, among which *a* ∈ [1,2*n*], the embedded *y*_1_ and *y*_2_. The formula (2) is no longer satisfied [9]. Like the general wavelet transform method, the wavelet low- and high-frequency coefficients obtained by the first level decomposition can be further decomposed into higher order.

### 2.3. Information Extraction and Original Image Restoration

At first, the receiver judges whether the pixel group meets the formula (2), if yes, it is the pixel group with embedded information; otherwise, the pixel group has no embedded information and is marked with a binary matrix *R*. For pixel groups with embedded information $\left({\tilde{y}}_{1},y_{2}\right)$, substitute it into formula (4) to calculate C. Then, the embedded information is recovered by using the corresponding relation. In this way, the recipient can extract additional information and auxiliary information *b*. Finally, the transformed pixel values are recovered, among which $y_{1}={\bar{y}}_{1}-a$, *y*_2_=*y*_2_, and the original pixels are recovered by inverse transformation, as shown in the following formula:

(5) $$\begin{array}{c} x_{1}=\frac{\left(n+1\right)y_{1}+n\left(y_{2}\right)}{2n+1},\\ x_{2}=\frac{\left(n+1\right)y_{2}+n\left(y_{1}\right)}{2n+1}. \end{array}$$

For a pixel group (*y*_1_, *y*_2_) satisfying formula (2), the original pixel value can be restored according to the corresponding auxiliary information *x*_1_=*y*_1_ − *b*, *x*_2_=*y*_2_. According to the above steps, the original image can be restored without loss [10].

Using the above algorithm, the plaintext image is subjected to decimal transformation, and two adjacent pixels are constrained by decimal transformation. According to the constraint relationship, the original image can be restored. However, after image encryption, the information concealer cannot find the transformed pixel group for information embedding by judgment, and the information cannot be reversibly embedded into the ciphertext image by addition. Therefore, in this study, the algorithm is improved, and a reversible information hiding algorithm for encrypted images based on decimal transformation is proposed. The specific algorithm will be given in the next section [11]. Ordinary wavelet transform cannot be used in reversible watermarking because it does not support complete reversibility. For example, in an 8-bit gray image, the pixel value is an integer between 0 and 255. The modification of coefficients after ordinary wavelet transform may cause the pixel value in the watermark image to be noninteger, which makes the implementation of reversibility difficult. In order to avoid these problems and ensure the reversibility of image transformation, reversible wavelet transform based on lifting scheme is adopted.

### 2.4. Histogram Translation

**Figure 1:**
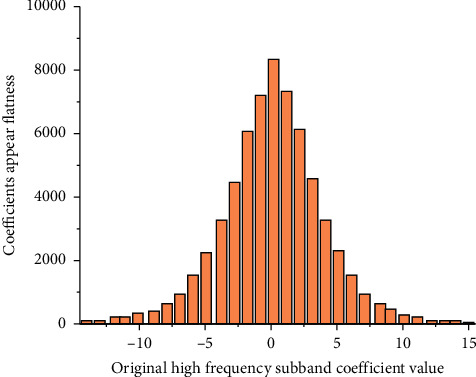
HH offspring coefficient histogram of gray image.

The histogram translation method is a common lossless data hiding method. The embedding principle of histogram translation method is to create a gap, which is the free space that can be used to embed data in the original image histogram or transform domain histogram. An example is given to illustrate the process of embedding data by the histogram method.  Figure 1 shows the histogram of HH subband coefficients of a gray image. Note that the coefficients of high-frequency subband are concentrated near the zero-value point [12].

**Figure 2:**
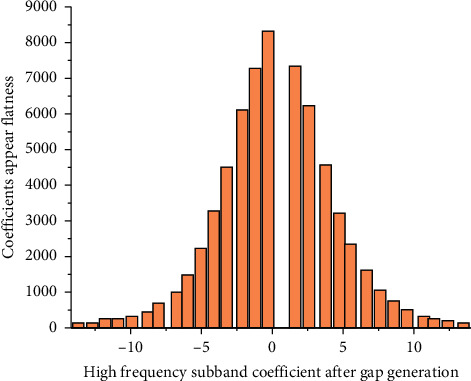
Schematic diagram of histogram slot.

In order to embed data where the coefficient value is *n*, first, an embedding gap is generated, and all coefficient values larger than *n* are increased by 1, as shown in Figure 2, where *n* = 0. After the gap is generated, the binary watermark message can be embedded in the free space. Of course, the coefficient can also be adjusted in the opposite direction, for example, an embedded watermark with a coefficient of −1 is selected. Then, all coefficients smaller than −1 are adjusted by an amplitude in the negative direction to generate a gap [13]. Ordinary wavelet transform cannot be used in reversible watermarking because it does not support complete reversibility. For example, in an 8-bit gray image, the pixel value is an integer between 0 and 255. The modification of coefficients after ordinary wavelet transform may cause the pixel value in the watermark image to be noninteger, which makes the implementation of reversibility difficult.

When embedding, scan coefficient values and all coefficients with coefficient values equal to 0 are coefficients that can be embedded with watermark. If the watermark to be embedded is 0, then the current coefficient remains unchanged. If the watermark to be embedded is 1, add 1 to the current coefficient. If the total amount of band embedded data is greater than the number of coefficients whose coefficient value is equal to *n*, we can continue to use other coefficient values to generate more free space. At the extraction end, all coefficients are scanned, and all coefficients with values equal to *n* or *n* + 1 contain watermark information. If the current coefficient value is equal to *n*, decode watermark bit 0. If the current coefficient value is equal to *n* + 1, decode watermark bit 1 and subtract 1 from the coefficient value to restore the original coefficient value. By this method, all the modified coefficients in the embedding process can be restored, and the original image can be restored without loss [14]. Two adjacent pixels are constrained by integer transformation of plaintext image. According to the constraint relation, the original image can be recovered. However, after the image is encrypted, the information hider cannot find the transformed pixel group through judgment to embed the information, and the information cannot be inserted reversibly into the ciphertext image through addition.

## 3. Introduction of Algorithm

**Figure 3:**
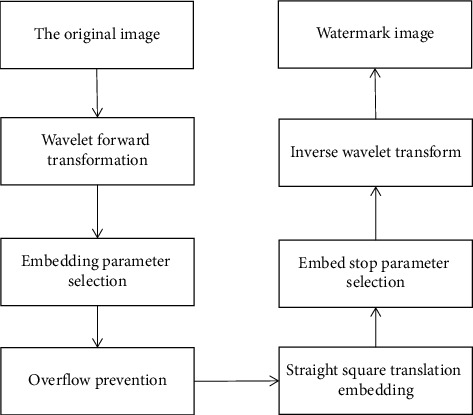
Algorithm embedding process diagram.

The embedding process of the digital image reversible watermarking algorithm based on fractional wavelet transform is shown in Figure 3. The embedding algorithm is mainly composed of 6 main modules, among which the forward transform and inverse transform of decimal-decimal wavelet transform the image from spatial domain to frequency domain and from frequency domain to spatial domain, respectively. The embedding parameter selection module is used for selecting the embedding position of the watermark. The antioverflow module is used to prevent serious image distortion caused by overflow in the embedding process and reduce the length of additional information. The histogram translation module is used for watermark embedding, and the embedding stop parameter selection module is used to control the embedding capacity and image distortion of the algorithm in the embedding process [15].

Embedding data on wavelet coefficients of an image by the histogram translation method will cause changes in pixel values in spatial domain, which may lead to inevitable overflow and underflow phenomena. Taking an 8-8 bit digital image as an example, overflow means that the pixel value exceeds 255, while underflow means that the pixel value is less than 0. Although, the pixel value greater than 255 will be truncated when the image is displayed. For example, the pixel value of 257 will suddenly change to 1, and a white pixel will suddenly change into a black one, which will cause salt pepper noise when the image is displayed and seriously affect the image quality. To prevent overflow, most solutions use location maps to indicate which coefficients cannot be changed, in order to pass the map to the decoding end [16].

In this study, a new method for generating maps is proposed, which makes full use of the characteristics of wavelet transform to reduce the problem of large size of coefficient maps. Wavelet transform shows good correlation and resolution in spatial domain and frequency domain of image, and its high-frequency subband carries spatial domain information of image. Because the overflow problem originates from those pixels at the boundary, it is possible to locate the corresponding coefficients in the frequency domain. After locating these special coefficients, in the embedding process, the map is referenced to exclude these pixels close to the boundary value. In order to explain the antioverflow method adopted in this study more clearly, an example is given. Assume that the original signal is a 4 × 4 two-dimensional array, and the values of the array are pixel values. Pixel values less than 10 and greater than 250 gray levels should be excluded in the embedding process [17].

## 4. Experimental Results and Analysis

First, Lena, an 8-bit grayscale image with a size of 512 × 512, is used as the original image for experiments. In the experiment, 3 common natural grayscale images (Lena, Baboon, and Peppers, all from the Internet, 512 × 512 × 8 bit depth, BMP format, 71DPI in horizontal and vertical resolution, and 257 KB in size) and 3 medical grayscale images (MRI_Skull, CT_Lung, and US _ WOMB, 512 × 512 × ) were randomly selected. The horizontal and vertical resolutions are 72DPI, and the size is 39.7 KB. The programming environment is the Matlab 7.0 and WindowsXP operating system (the main frequency is 1.6 GHz and the memory is 1.25 GB) [18].

The watermark embedding capacity is measured by bpp, and the embedded watermark information is generated by rand () random function in Matlab. The PSNR calculation formula for measuring image distortion is as follows:

(6) $$\begin{array}{c} \text{PSNR}=10\;\log_{10}\left(\frac{\text{NM}{\left(2^{P}-1\right)}^{2}}{\sum_{i,j=1,1}^{NM}{\left(I\left(i,j\right)-I_{w}\left(i,j\right)\right)}^{2}}\right), \end{array}$$

where *M*, *N*, *P*, *n*, and *p* represent the width, height, and bit depth of the image, and *I*(*i*, *j*) and *I*_*w*_(*i*, *j*) represent the pixel values of the original image and watermark image, respectively.

### 4.1. Algorithm Complexity Analysis

The computational complexity of this algorithm depends on the operations of wavelet transform and histogram shift. The time complexity of standard discrete wavelet transform is *O*(*N*), computational complexity of histogram translation, the number of input pixels is *N*, and translation times are *k*_1_. Directly, the time complexity is *O*(*k*_1_ × *N*) [18].

### 4.2. Experimental Results

**Figure 4:**
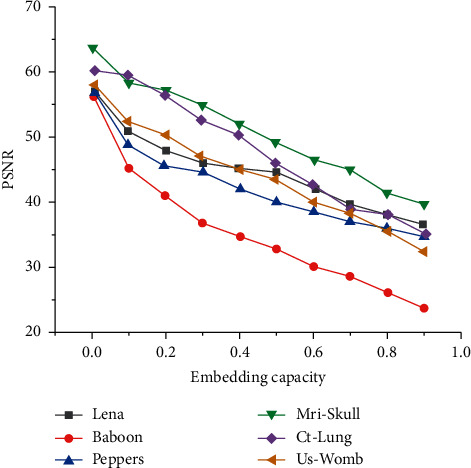
PSNR test results of different carrier images under different embedding capacities.

Figure 4 shows the PSNR test results of different carrier images under different embedding capacities.

It can be seen from Figure 4 that, first of all, except for Baboon image, other images can keep good watermark image quality when the embedding capacity is close to 1 bpp, and the PSNR value is above 30 dB. Second, the actual embedding performance of medical images is obviously better than that of natural images. Because of the characteristics of medical images, the histogram of wavelet coefficients is more concentrated at point 0 and still keeps Laplace distribution. In order to achieve higher embedding capacity, multilayer embedding strategy can be adopted. In the multilayer embedding strategy, the watermark image is used to perform the embedding process many times to embed more data. This shows that a small amount of histogram translation can produce high watermark embedding space and bring smaller distortion, which is superior to natural images. In addition, the same type of images have obvious differences in embedding performance due to their different texture features. For example, the texture of Baboon image is the most complex, and its high-frequency coefficient histogram is relatively evenly distributed. As a result, more coefficients are used to embed data, which has the lowest PSNR value compared with other natural images in the experiment. Among the current algorithms, few algorithms can achieve a single-layer embedding capacity of 1 bpp in image carriers like Baboon. In medical images, the texture of US_Womb image is the most complex, the correlation between adjacent pixels is poor, and its embedding performance is the worst, which is close to general natural images such as Lena. Because the data are embedded in the high-frequency wavelet subband, each watermark image has high-frequency noise, and because of the existence of higher high-frequency coefficients, it is sharper than the original image, but even in the case of lower PSNR value, it does not have a serious impact on the visual quality of the image. On the whole, the distortion performance under each load capacity depends on the characteristics of each image because the number of high-frequency and low-frequency coefficients of each image is different, and the shape of gray histogram affects the coefficient mapping matrix in the process of preventing overflow. In addition, because medical images usually have a bit depth of 12 bits, this work studies the influence of image bit depth on our algorithm. Through experimental analysis, it is found that the embedding capacity of the same MRI_Skull medical image is much higher than that of the image with the depth of 8 bits because when the image pixels are expressed with the depth of 12 bit, the highest pixel value reaches 4095, so the general image pixel value will not use such a large space, and the pixels at the overflow edge are greatly reduced, thus reducing the length of additional information. In addition, after wavelet decomposition, the number of high-frequency subband coefficients near zero value greatly increases, which increases the embedding capacity [19].

## 5. Conclusion

This study presents a reversible watermarking algorithm for digital images based on fractional wavelet transform and histogram shift. The watermark is embedded by using the high-frequency coefficients after fractional wavelet decomposition. The algorithm of selecting embedding parameters ensures high embedding capacity, and the algorithm of selecting embedding stop parameters ensures the fidelity of watermark image. In addition, in order to effectively prevent overflow in the embedding process, a new location mapping method is proposed. Experimental results show that, except Baboon image, other images can keep good watermark image quality when the embedding capacity is close to 1 bpp, and the PSNR value is above 30 dB. Second, the actual embedding performance of medical images is obviously better than that of natural images. The histogram of wavelet coefficients is more concentrated at point 0 and still keeps Laplace distribution. Only by possessing both the changing key and the embedding key can the embedded information be correctly extracted from the ciphertext image. Only when the receiver has both the transformation key and the encryption key, can the receiver restore the original image without loss. After experimental comparison, the embedding rate of this algorithm is higher; while ensuring large embedding capacity, it can strengthen the protection of embedded information, which is of certain significance to the management of ciphertext data.

## Data Availability

The data used to support the findings of this study are available from the corresponding author upon request.
